# Assessment of pharmacodynamic interactions of two-drug combinations of five selected cytostatics in an in vitro model of human melanoma: an isobolographic analysis

**DOI:** 10.1007/s00210-025-04712-9

**Published:** 2025-11-12

**Authors:** Paula Wróblewska-Łuczka, Agnieszka Góralczyk, Jarogniew J. Łuszczki

**Affiliations:** Department of Occupational Medicine, Medical University of Lublin, Lublin, Poland

**Keywords:** Melanoma, Isobolographic analysis, Chemotherapeutics, Cisplatin, Mitoxantrone, Docetaxel, Vemurafenib, Selumetinib

## Abstract

Melanoma ranks 17th among the most frequently diagnosed cancers, with an upward trend in incidence. Treatment of this cancer is based on surgical removal of the lesion, but when metastases or recurrence occur, immunotherapy or chemotherapy is used. Most of the drugs used are characterized by numerous side effects or drug resistance that appears after some time of use. Therefore, multi-drug therapy is more often considered. The aim of the study was to assess the nature of the pharmacodynamic interaction of five different cytostatics (in a fixed dose ratio of 1:1) using a mathematical-statistical method—isobolographic analysis. The experiments were conducted on four human malignant melanoma cell lines (A375, SK-MEL28, FM55M2, FM55P) and studied all possible (ten) pairs of combinations of five standard cancer chemotherapy drugs (based on the MTT test): cisplatin, mitoxantrone, docetaxel, vemurafenib, and selumetinib. Our experiments showed that the most advantageous combination was the combination of vemurafenib with docetaxel for human melanoma lines—statistically significant synergy interaction for three cell lines. Also noteworthy are the combinations: cisplatin with mitoxantrone, vemurafenib with selumetinib, and vemurafenib with mitoxantrone. From a therapeutic point of view, the worst combination is cisplatin with docetaxel and cisplatin with selumetinib (interaction with a tendency to antagonism for the two cell lines). Monotherapy for melanoma does not produce good results, so the best drug combinations are sought, which would improve long-term, durable patient response and overcome drug resistance. It would also be good if the combination used caused fewer side effects than monotherapy, and this could be achieved in the case of using synergistic combinations.

## Introduction

According to the Global Cancer Observatory data from 2020, melanoma ranks 17th among the most commonly diagnosed cancers in the world. While some cancers, especially in developed countries, are experiencing a downward trend in incidence, epidemiological data on malignant melanoma indicate a sustained increase in incidence, which poses a serious public health challenge (Sung et al. 2021).

Melanoma is the most common in highly developed countries, especially in light-skinned populations. Differences in melanoma incidence are related to the level of UVR (ultraviolet radiation) exposure, which depends on geographical latitude (Armstrong and Kricker 2001). The risk of developing melanoma is the result of the interaction of both genetic and environmental factors. Despite the complexity of these interactions, increased exposure to UVR is considered a key risk factor for melanoma (Garland et al. 1993).

The main method of treating melanoma is surgery, and in the case of metastases, chemo- and radiotherapy are used. Understanding the mechanisms and cellular pathways has allowed the development of more effective therapies. These include immune checkpoint inhibitors, targeted therapies based on signaling pathways, intralesional therapy (for irremovable changes), and local therapy (for single changes) (Garbe et al. 2011).

Many of the cytostatic drugs used are characterized by numerous side effects or drug resistance that appears after some time of use (Chen et al. 2022; Garbe and Eigentler 2018). For these reasons, multidrug therapy for melanoma is increasingly being considered. The aim of our experiment was to assess the nature of the pharmacodynamic interactions for two-drug combinations (in a fixed 1:1 dose ratio) of five chemotherapeutic agents with different mechanisms of action: cisplatin, mitoxantrone, docetaxel, vemurafenib, and selumetinib, using an isobolographic analysis, which is a mathematical-statistical method allowing for determining pharmacodynamic interactions between drugs. This method has been used many times to assess the interactions of various chemical substances on cancer cells (Wawruszak et al. 2019; Jarząb et al. 2014; Wróblewska-Łuczka et al. 2021).

Cisplatin is considered the gold standard in experimental in vitro studies due to the reference comparison of the antitumor activity of the compound tested in the study. In the case of cisplatin, the drug binds to genomic and mitochondrial DNA, forming platinum adducts with DNA and blocking DNA replication, which consequently leads to cell necrosis or apoptosis (Achkar et al. 2018). Mitoxantrone is an anthraquinone anticancer drug that inhibits DNA synthesis and transcription (Legut et al. 2014). This compound intercalates in DNA, leading to strand breaks, inhibition of DNA synthesis and transcription, and consequently to cell apoptosis (Lenk et al. 1987). Docetaxel, as one of the taxanes, stops cell division due to an abnormal amount of genetic material in the cell (docetaxel stabilizes microtubules) (Goodson and Jonasson 2018). It mainly acts on the G1/S point dependent on the p53 protein (Yasuhira et al. 2016). Vemurafenib is able to inhibit mutant BRAF serine-threonine kinase and selectively binds to the ATP-binding site of BRAF V600E kinase and inhibits its activity. Vemurafenib potently inhibits ERK phosphorylation and cell proliferation with BRAF mutation only (Garbe and Eigentler 2018). The last drug used in the study was selumetinib—a MEK inhibitor. Selumetinib acts on the mitogen-activated kinase signaling pathway (MAPK) containing RAS/RAF/MEK/ERK kinases. Selumetinib is an intracellular relay pathway that transmits mitogenic signals to the cell nucleus through a series of phosphorylation of the molecules that make up this pathway. MEK inhibitors increase the sensitivity of cells to traditionally used chemotherapeutics, such as cisplatin or taxoids. MEK inhibitors are active in cells with a confirmed BRAF mutation (Inamdar et al. 2010; Smalley and Flaherty 2009).

The above-mentioned chemotherapeutic agents were selected due to their various and different molecular mechanisms of action, which may have a beneficial effect on the resulting drug interactions, which would be desirable in the anticancer therapy.

## Materials and methods

### Malignant melanoma cell culture

In this experiment, two metastatic melanoma cell lines (FM55M2 and SK-MEL28) and two primary (FM55P and A375) were used. From the European Collection of Authenticated Cell Cultures (ECACC), we purchased FM55P and FM55M2 cell lines, and from the American Type Culture Collection (ATCC), we purchased A375 and SK-MEL28 cell lines. Cell culture conditions were as follows: 37 °C in a humidified atmosphere of 95% air and 5% CO_2_. Culture media used are as follows: for A375, DMEM high glucose; for SK-MEL28, EMEM, and for FM55P and FM55M2, RPMI1640. Previous publications have described in detail the growth conditions of cell line cultures (Wróblewska-Łuczka et al. 2021, 2022a).

### Tested drugs

The examined chemotherapeutics—mitoxantrone, docetaxel, vemurafenib, and selumetinib—were dissolved in DMSO, whereas cisplatin was dissolved in PBS buffer (all the investigated drugs were of chemical purity from Sigma-Aldrich, St. Louis, MO, USA). Compounds were dissolved to the tested concentration in the growth medium before adding compounds to the microtiter (96-well) plate. DMSO had no effect on cell growth (safe concentration of DMSO maintained at 0.1%).

### Cell viability assessment—MTT test

The influence of five chemotherapeutics (cisplatin, mitoxantrone, docetaxel, vemurafenib, and selumetinib) on cell viability was assessed using the MTT assay. Four melanoma lines (density 2–3 × 10^4^ cells/ml depending on the cell line) were placed on microtiter plates (NEST Biotechnology, Jiangsu, China). After 24 h of incubation, the medium was removed, and fresh medium was added with the increasing concentrations of tested drugs (cisplatin, mitoxantrone, docetaxel, vemurafenib, and selumetinib). The steps of the MTT assay have been described earlier (Wróblewska-Łuczka et al. 2022a; Marzęda et al. 2022). Each experiment in the MTT assay was performed in triplicate to ensure repeatability of results.

### Isobolographic analysis

To thoroughly characterize pharmacodynamic interactions between two-drug combinations of five cytostatics in the malignant melanoma cell lines (FM55P, A375, FM55M2, and SK-MEL28), we used the isobolographic analysis in the MTT assay. Based on the MTT test, the percentage of inhibition of cell viability in relation to the specific concentrations of the tested drugs (cisplatin, mitoxantrone, docetaxel, vemurafenib, and selumetinib) was determined. Due to log-probit analysis (Litchfield and Wilcoxon 1949 Jun), concentration–response effect graphs were drawn, and the log-probit concentration–response lines allowed for calculating the median inhibitory concentrations (IC_50_ values) for every tested drug. Collateralism of two concentration–response curves was confirmed by means of the test for parallelism (i.e., vemurafenib + cisplatin, vemurafenib + mitoxantrone, vemurafenib + docetaxel, vemurafenib + selumetinib, selumetinib + cisplatin, selumetinib + mitoxantrone, selumetinib + docetaxel, cisplatin + mitoxantrone, cisplatin + docetaxel, and mitoxantrone + docetaxel), as described earlier (Wróblewska-Łuczka et al. 2021; Luszczki 2007). From the experimentally denoted IC_50_ values (based on MTT assay) for the drugs administered alone, median additive inhibitory concentrations for the mixtures (IC_50 add_) (at the fixed-ratio of 1:1) were calculated, as described previously (Tallarida et al. 1989). More information on isobolographic analysis can be found elsewhere (Marzęda et al. 2022; Tallarida 2011 Nov). In the final stage, polygonograms were drawn to illustrate and summarize the types of interactions between two-drug combinations of five cytostatics in human melanoma cells. In each graph, drugs from combinations are connected by a line. The color of the line symbolizes the interaction: black, additive; green, synergistic; and red, antagonistic interaction, as reported elsewhere (Chou 2008).

### Statistical analysis

GraphPad Prism 8.0 Statistic Software (San Diego, CA, USA) was used for statistical analysis of data (from the MTT test). The calculations were performed with the one-way ANOVA test followed by Tukey’s post-hoc test. Every data was presented as means ± standard errors (SEM) (*p < 0.05, **p < 0.01, ***p < 0.001, ****p < 0.0001). The median inhibitory concentration (IC_50_ and the IC_50 mix_) values for five tested cytostatics (cisplatin, mitoxantrone, docetaxel, vemurafenib, and selumetinib) were computed with the log-probit method, as described earlier (Wróblewska-Łuczka et al. 2021; Litchfield and Wilcoxon 1949 Jun; Luszczki 2007). The unpaired Student’s *t*-test with Welch’s correction was used to compare the experimentally-derived IC_50 mix_ values (for the two-drug mixtures of five chemotherapeutics with their respective, theoretically calculated and presumed to be additive IC_50 add_ values, as recommended elsewhere (Tallarida 2011 Nov).

## Results

### Cell viability assessment—MTT assay results

**Fig. 1:**
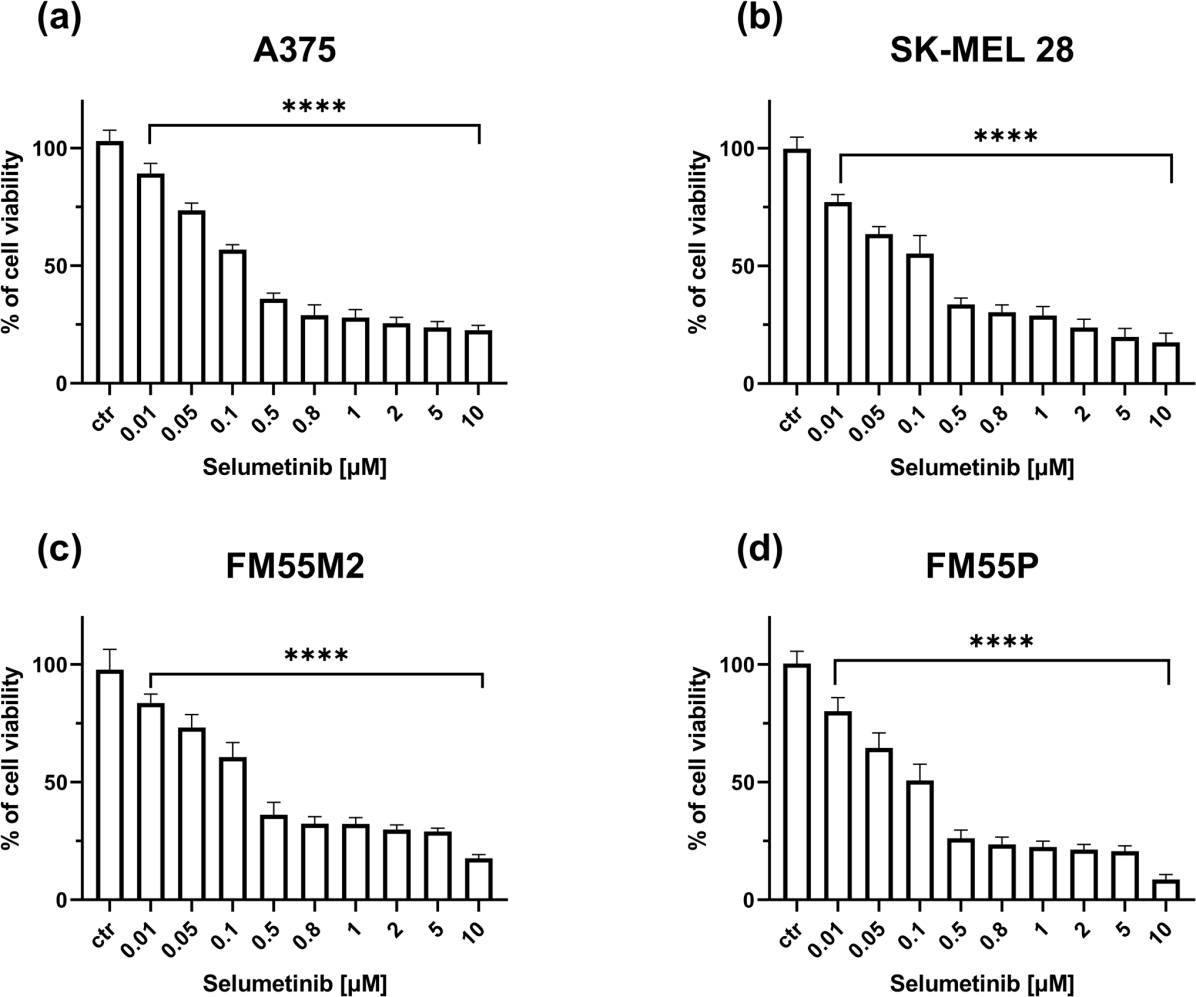
Impact of selumetinib on cell viability in the MTT test in A375 (**a**), SK-MEL28 (**b**), FM55M2 (**c**), and FM55P (**d**) cell lines. Columns represent mean ± SEM (****p < 0.0001 vs. the control (ctr) group)

All the tested drugs—cisplatin, mitoxantrone, docetaxel, vemurafenib, and selumetinib—inhibited melanoma cell viability in a concentration-dependent manner. The effect of selumetinib on melanoma cell viability is shown in Fig. 1.

### Pharmacodynamic interactions for a two-drug combination of five chemotherapeutics: cisplatin, mitoxantrone, docetaxel, vemurafenib, and selumetinib

In the next stage of our research, the inhibitory effect of cisplatin, mitoxantrone, docetaxel, vemurafenib, and selumetinib in two-drug combinations on the viability of malignant melanoma cells was analyzed. The aim was to determine whether any of the combinations was characterized by the desired synergistic interaction. Concentration–response curves (CRRC) were determined according to the log-probit method (Litchfield and Wilcoxon 1949 Jun). The equations of the curves were determined for the compounds used alone and for the two-drug combinations of cisplatin, mitoxantrone, docetaxel, vemurafenib, and selumetinib, which allowed to determine the median inhibitory concentrations (IC_50_ values ± SEM). In our previous experiments, we determined the mean inhibitory concentrations (IC_50_) for cisplatin (1.3 to 3.3 µM) (Wróblewska-Łuczka et al. 2021, 2022b; Marzęda et al. 2022), mitoxantrone (0.04 to 1.7 µM) (Marzęda et al. 2022; Wroblewska-Łuczka et al. 2022b), docetaxel (from 1.27 to 15.83 nM) (Wróblewska-Łuczka et al. 2022a), and vemurafenib (0.25 to 6.07 µM) (Wróblewska-Łuczka et al. 2023) depending on the cell line. The IC_50_ of the tested selumetinib was determined at 0.1 µM for the FM55P cell line, 0.2 µM for the A375 line, 0.15 µM for the SK-MEL28 line, and 0.22 µM for the FM55M2 cell line. All the IC_50_ values are presented in Table 1.

**Table 1:** Summary of experimentally determined IC_50_ values of the tested chemotherapeutics against four lines of human malignant melanoma. Results obtained based on MTT tests, value IC_50_ ± SEM

Cell line IC_50_of tested drug	FM55P	A375	FM55M2	SK-MEL28	References
Cisplatin (µM)	1.49 ± 0.30	1.29 ± 0.34	1.70 ± 0.30	3.30 ± 0.70	Wróblewska-Łuczka et al. 2021; Marzęda et al. 2022; Wróblewska-Łuczka et al. 2022b)
Docetaxel (nM)	1.27 ± 0.55	15.05 ± 3.27	2.06 ± 0.66	15.83 ± 9.05	Wróblewska-Łuczka et al. 2022a)
Mitoxantrone (µM)	0.35 ± 0.10	0.04 ± 0.02	0.13 ± 0.02	1.74 ± 0.51	Marzęda et al. 2022; Wróblewska-Łuczka et al. 2022b)
Vemurafenib (µM)	0.76 ± 0.26	6.07 ± 2.06	0.62 ± 0.27	0.25 ± 0.13	Wróblewska-Łuczka et al. 2023)
Selumetinib (µM)	0.1 ± 0.04	0.2 ± 0.07	0.22 ± 0.08	0.15 ± 0.07	This study

In the next step, the test of parallelism of the experimentally determined curves for all tested combinations was performed. Parallelism or its lack affects the appearance of the additivity line in the isobolograms.

**Fig. 2:**
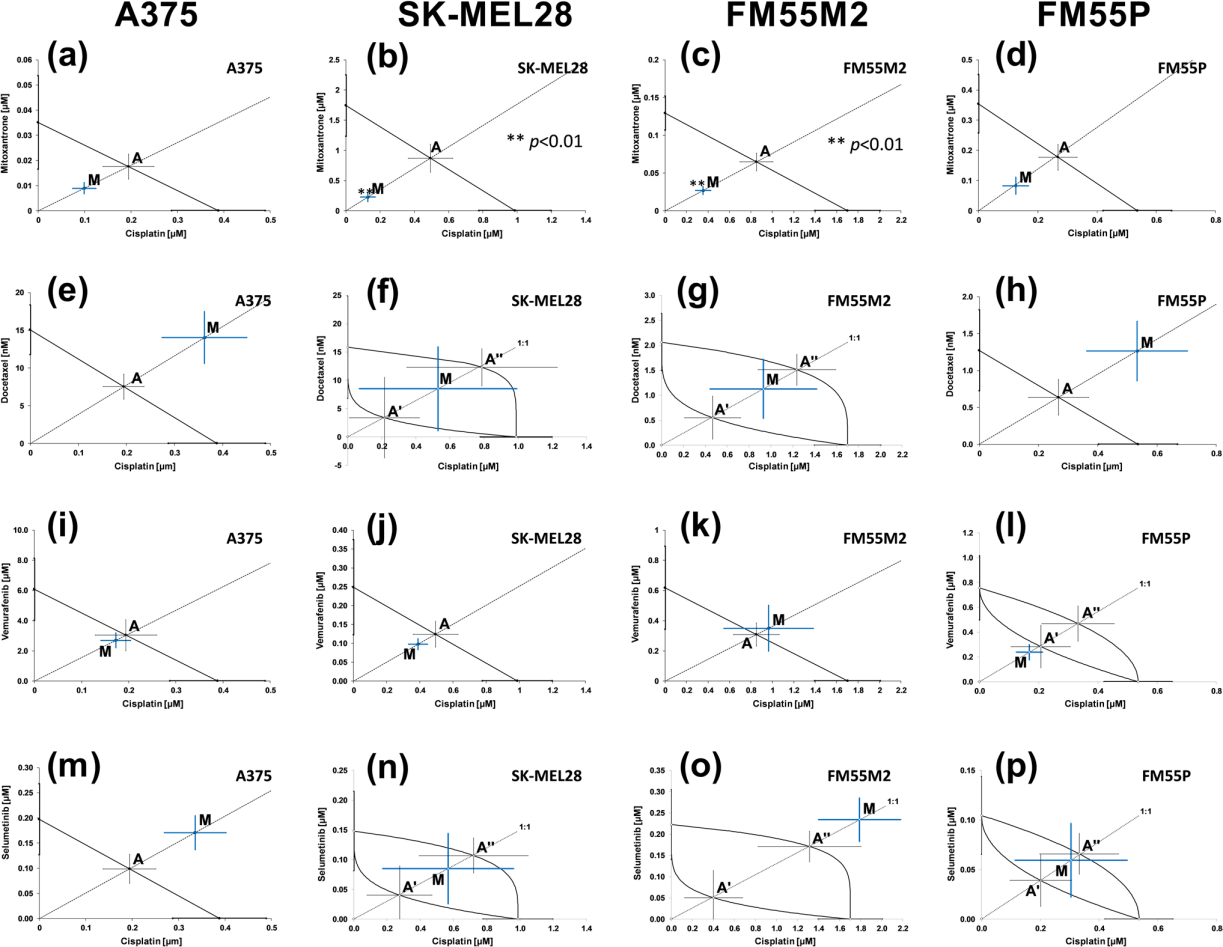
Interactions between cisplatin and mitoxantrone (**a**–**d**), cisplatin and docetaxel (**e**–**h**), cisplatin and vemurafenib (**i**–**l**), and cisplatin and selumetinib (**m**–**p**) with their anti-proliferative effects on A375 (**a**, **e**, **i**, **m**), SK-MEL28 (**b**, **f**, **j**, **n**), FM55M2 (**c**, **g**, **k**, **o**), and FM55P (**d**, **h**, **l**, **p**) cell lines. IC_50_ (± SEM) values for cisplatin, mitoxantrone, docetaxel, vemurafenib, and selumetinib are placed in the Cartesian system of coordination. Points A, A′, A″—theoretically calculated IC_50add_ values (± SEM). Point M—experimentally derived IC_50mix_ value (± SEM). **p < 0.01 (Student’s *t*-test with Welch’s correction)

The conducted experiments and isobolographic analysis for the combination of cisplatin + mitoxantrone (at the fixed-ratio of 1:1) showed a statistically significant synergistic interaction for the SK-MEL28 and FM55M2 cell lines (Fig. 2b and c) and the additive interaction with a tendency to synergy for A375 and FM55P cell lines (Fig. 2a and d). Cisplatin with vemurafenib showed an additive interaction for all tested melanoma cell lines (Fig. 2i, j, k, and l). For the combination of cisplatin + docetaxel and cisplatin + selumetinib, we observed an additive interaction, although for the A375 cell line in both combinations, an additive interaction with a tendency to antagonism was observed (Fig. 2e and m). A tendency to antagonism was also observed in the case of cisplatin + docetaxel for the FM55P cell line (Fig. 2:h) and cisplatin + selumetinib for the FM55M2 cell line (Fig. 2o).

**Fig. 3:**
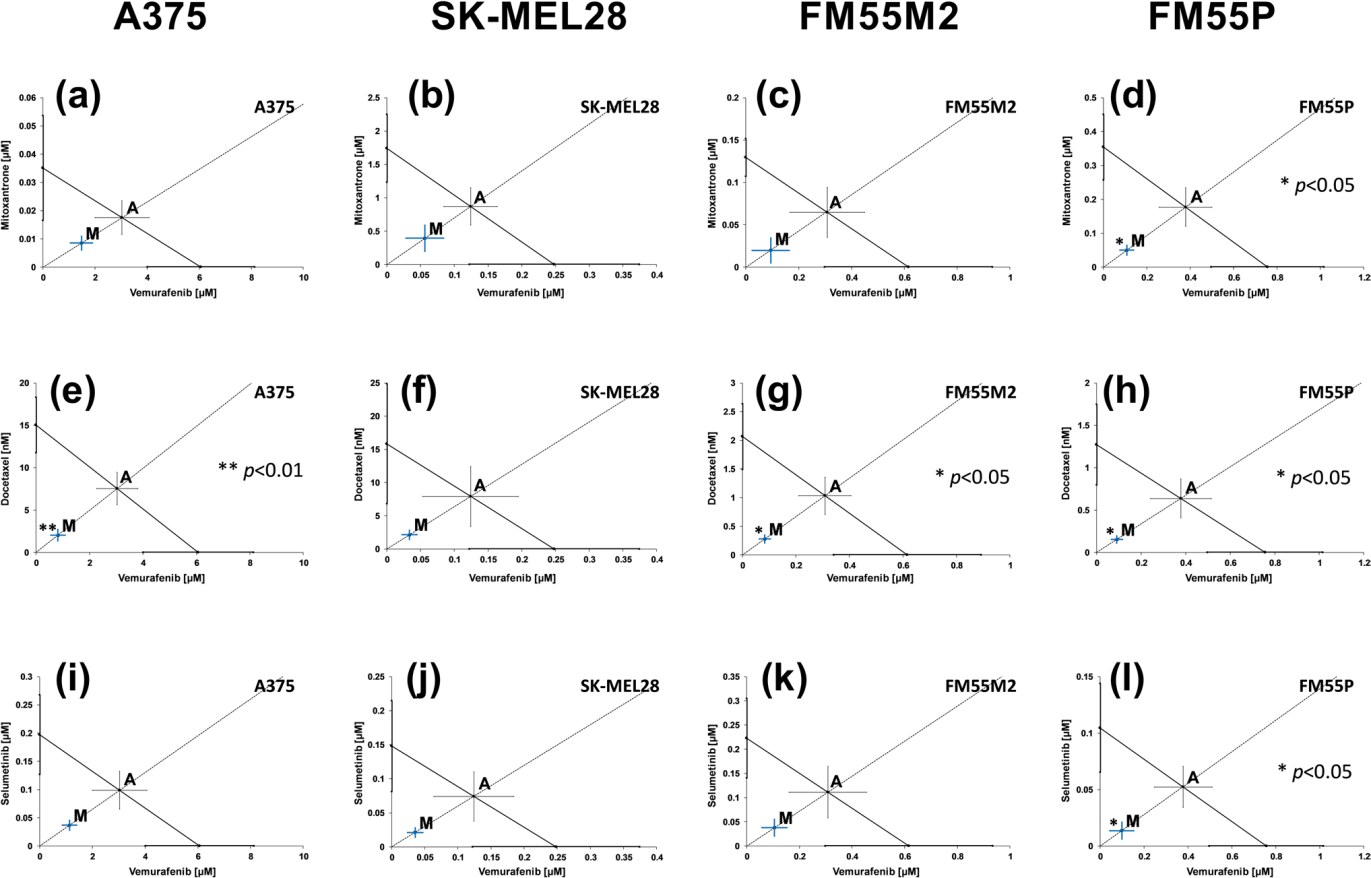
Interactions between vemurafenib and mitoxantrone (**a**–**d**), vemurafenib and docetaxel (**e**–**h**), and vemurafenib and selumetinib (**i**–**l**) with their anti-proliferative effects on A375 (**a**, **e**, **i**), SK-MEL28 (**b**, **f**, **j**), FM55M2 (**c**, **g**, **k**), and FM55P (**d**, **h**, **l**) cell lines. IC_50_ (± SEM) values for mitoxantrone, docetaxel, vemurafenib, and selumetinib are placed in the Cartesian system of coordination. Points A, A′, A″—theoretically calculated IC_50add_ values (± SEM). Point M—experimentally derived IC_50mix_ value (± SEM). **p < 0.01 and *p < 0.05 (Student’s *t*-test with Welch’s correction)

Combinations of vemurafenib with mitoxantrone, vemurafenib with docetaxel, and vemurafenib with selumetinib (at the fixed-ratio of 1:1) showed the additive interaction with a tendency to synergy (Fig. 3) and statistically significant synergistic interaction for the A375 (Fig. 3e, vemurafenib + docetaxel), FM55M2 (Fig. 3g, vemurafenib + docetaxel), and FM55P cell lines in every of three combinations, respectively (Fig. 3d, h, and l).

**Fig. 4:**
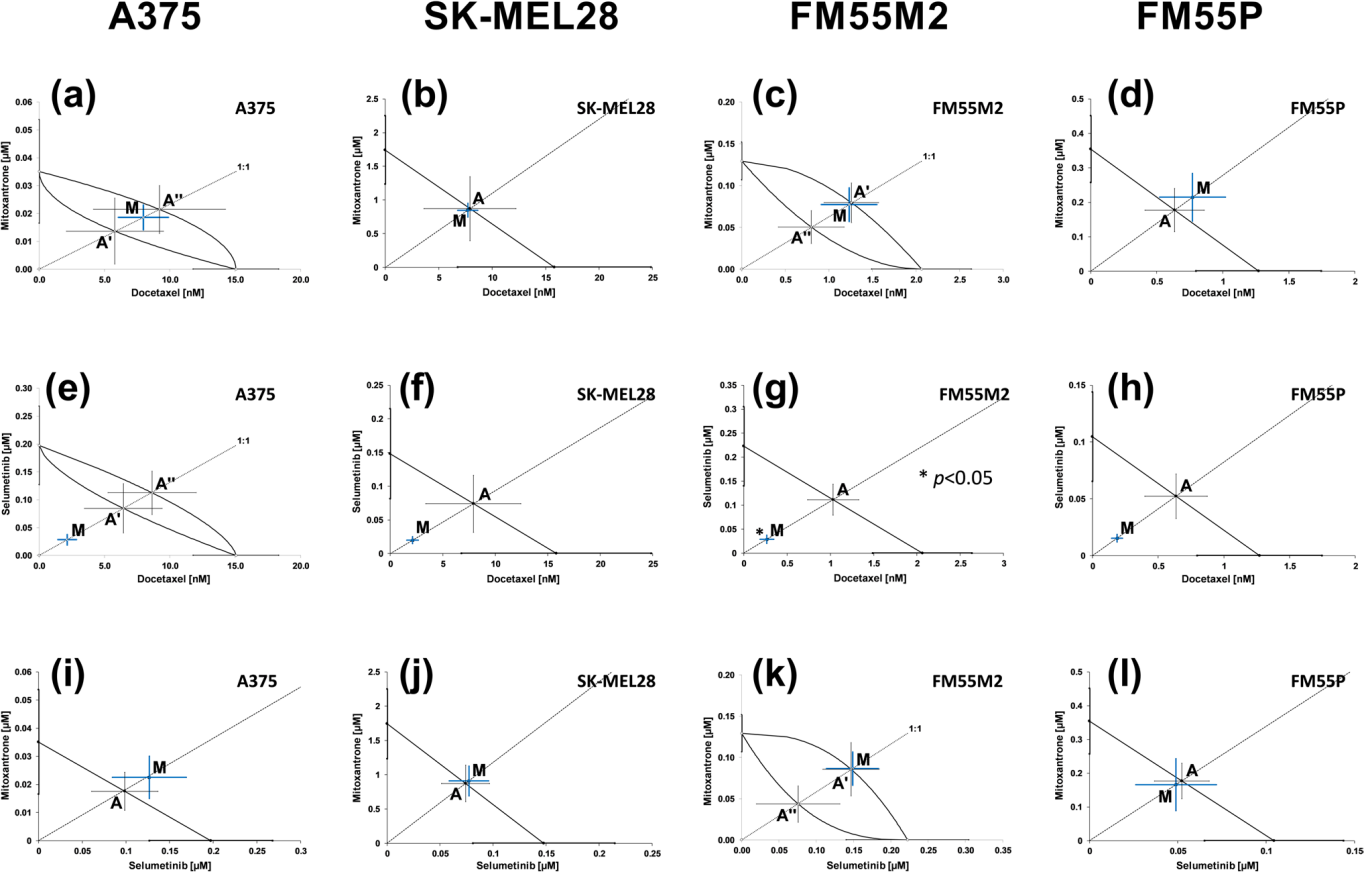
Interactions between docetaxel and mitoxantrone (**a**–**d**), docetaxel and selumetinib (**e**–**h**), and selumetinib and mitoxantrone (**i**–**l**) with their anti-proliferative effects on A375 (**a**, **e**, **i**), SK-MEL28 (**b**, **f**, **j**), FM55M2 (**c**, **g**, **k**), and FM55P (**d**, **h**, **l**) cell lines. IC_50_ (± SEM) values for mitoxantrone, docetaxel, and selumetinib are placed in the Cartesian system of coordination. Points A, A′, A″—theoretically calculated IC_50add_ values (± SEM). Point M—experimentally derived IC_50mix_ value (± SEM). *p < 0.05 (Student’s *t*-test with Welch’s correction)

The isobolographic analysis for the combination of docetaxel with mitoxantrone (Fig. 4a, b, c, and d) and selumetinib with mitoxantrone (Fig. 4i, j, k, and l) (at the fixed-ratio of 1:1) showed the additive interaction for the tested melanoma cell lines. For the combination of docetaxel with selumetinib, we observed the additive interaction with a tendency to synergy for A375, SK-MEL28, and FM55P cell lines (Fig. 4e, f, and h) and statistically significant synergistic interaction for the FM55M2 cell line (Fig. 4g).

**Fig. 5:**
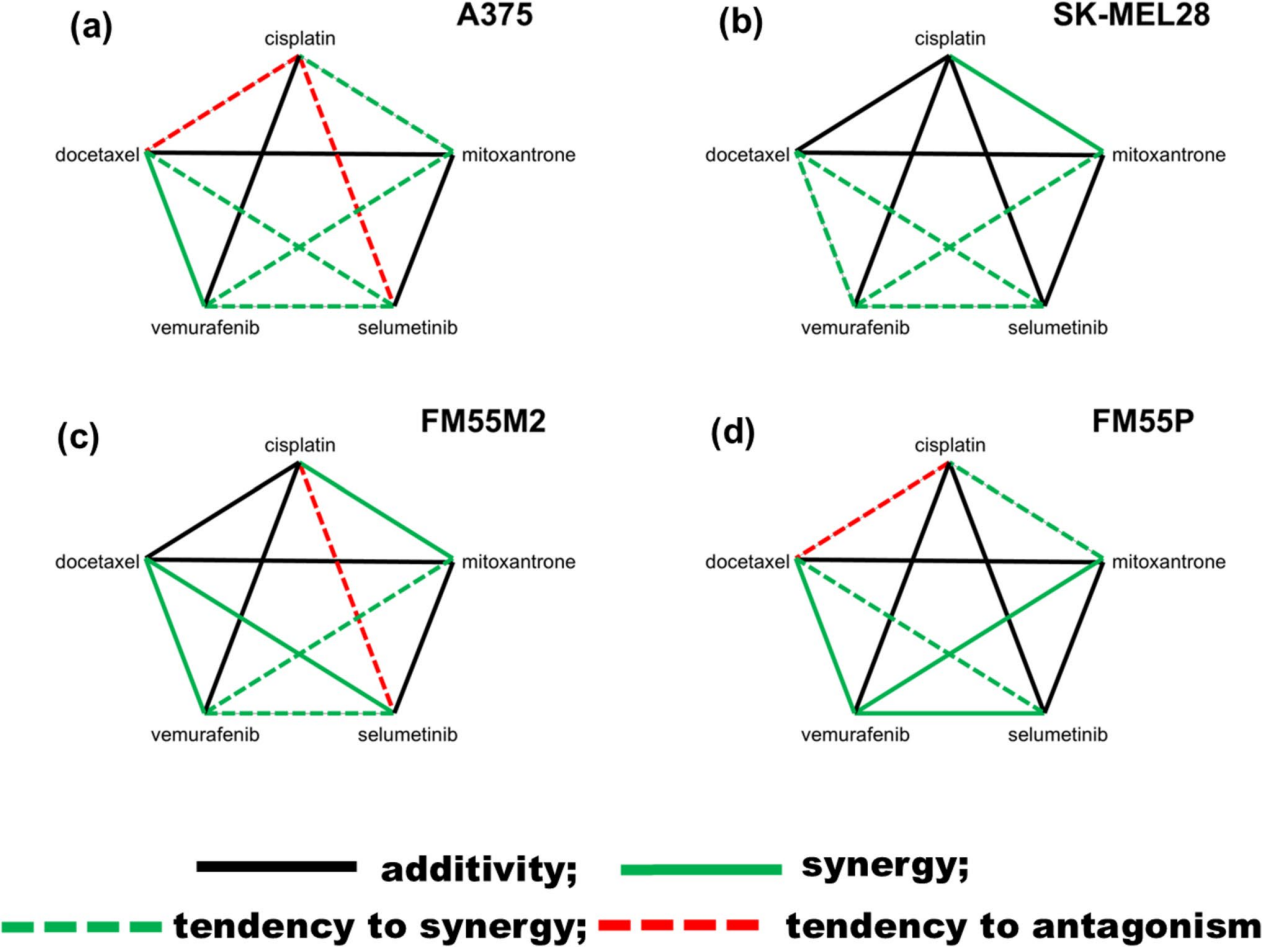
Polygonograms illustrating the interactions for two-drug mixtures of cisplatin, mitoxantrone, docetaxel, vemurafenib, and selumetinib in in vitro tests for four malignant melanoma cell lines: A375 (**a**), SK-MEL28 (**b**), FM55M2 (**c**), and FM55P (**d**). Black lines illustrate the additive interactions, whereas the green lines indicate synergistic and red lines antagonistic interactions. The dashed lines indicate a tendency toward a given interaction

**Fig. 6:**
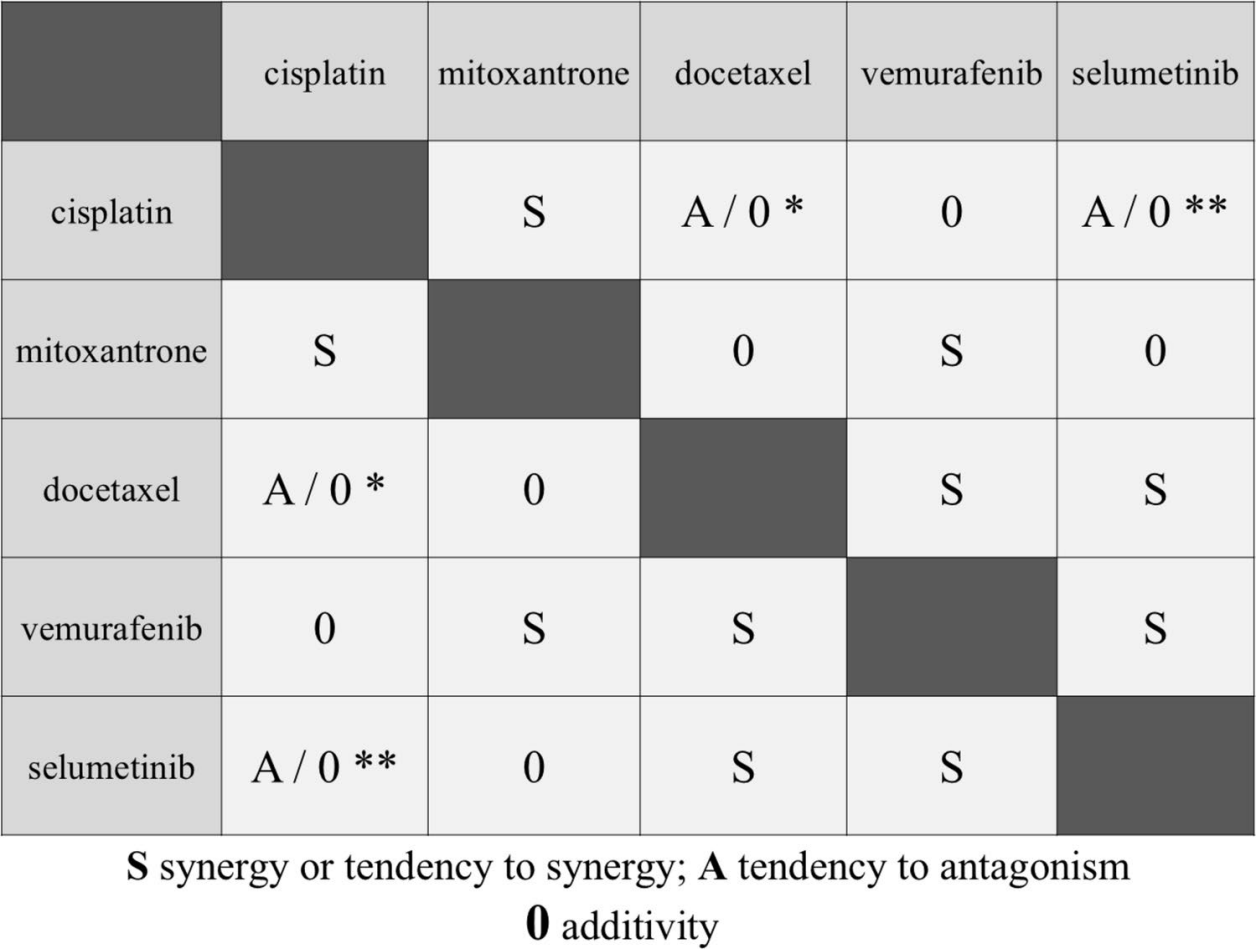
Table matrix summarizing the interactions for two-drug mixtures of cisplatin, mitoxantrone, docetaxel, vemurafenib, and selumetinib in in vitro tests for four malignant melanoma cell lines. “S” means synergy or tendency to synergy interaction, “A” means tendency to antagonism, and “0” means additive interaction for all tested cell lines. Exceptions: the asterisk symbol (*) means interaction with a tendency to antagonism for the cell lines A375 and FM55P and addition for the lines: SK-MEL28 and FM55M2, and the asterisk symbols (**) mean interaction with a tendency to antagonism for the cell lines A375 and FM55M2 and addition for the lines: SK-MEL28 and FM55P

A polygonogram (Fig. 5) and matrix table (Fig. 6) were drawn to graphically summarize the interactions for the two-drug mixture of five cytostatics (cisplatin, mitoxantrone, docetaxel, vemurafenib, and selumetinib). Presenting the interactions in this form makes it easier to evaluate the obtained interactions between drugs, which allows for faster determination of the most advantageous combinations. Our experiments showed that the most advantageous combination was the combination of vemurafenib with docetaxel for human melanoma lines with statistically significant synergistic interaction for three cell lines (Figs. 5a, c, and d and 6). Also, the combinations of cisplatin with mitoxantrone (synergy for two lines and a tendency towards synergy for the other two), vemurafenib with selumetinib, and vemurafenib with mitoxantrone (synergy for one cell line and a tendency towards synergy for the other three cell lines) are worthy of mention. From a therapeutic point of view, the worst combination is cisplatin with docetaxel and cisplatin with selumetinib (displaying additive interaction with a tendency to antagonism for the two cell lines) (Figs. 5 and 6).

## Discussion

Many of the cytostatic drugs used are characterized by numerous side effects or drug resistance that appears after some time of use. For example, vemurafenib (the BRAF inhibitor) is an effective cytostatic drug used in the treatment of melanoma (due to frequent BRAF V600E mutations, detected in about 40% of patients), but resistance is observed after 6–10 months of therapy. Vemurafenib therapy is associated with side effects such as joint pain, headaches, and fever. It is worrying that in some patients after monotherapy with this drug, other skin cancers appear: squamous cell carcinoma or keratoacanthoma (Chen et al. 2022; Garbe and Eigentler 2018). The use of mitoxantrone is also associated with side effects such as bone marrow suppression or cardiotoxicity (Legut et al. 2014 ), and cisplatin therapy is associated with its strong nephrotoxicity, neurotoxicity, and hepatotoxicity (Oun et al. 2018 Jun 12). Chemotherapy resistance is a common cause of cancer treatment failure. It is estimated to be responsible for more than 90% of deaths in cancer patients receiving traditional chemotherapeutic agents and/or new targeted drugs (Bukowski et al. 2020). For these reasons, multidrug therapy for melanoma is increasingly being considered.

From a pharmacological point of view, three main types of interactions between anticancer drugs can be distinguished, including subadditivity (antagonism), additivity, and supraadditivity (synergy). The method of choice is the isobolographic analysis, which allows for precise classification of the type of interactions observed experimentally between two or more drugs used in a mixture (Luszczki 2007; Tallarida et al. 1989).

Combinations of drugs exhibiting antagonistic interactions in terms of the anticancer activity are not recommended for further studies and clinical application, because the drugs would have to be used in higher doses to achieve the elimination of 50% of cancer cells. In such a case, in clinical conditions, patients should receive higher doses of drugs, and therefore, adverse effects may occur more often than expected. As for the mechanism of action of drugs exhibiting antagonistic interaction, one of the drugs in its anticancer activity probably competes with the other drug and reduces its effect. Another explanation is also possible, taking into account the fact that one drug can affect different phases of the cell cycle. In such a case, one of the drugs used in the mixture stops/blocks the cell cycle, rendering the other drug ineffective, especially when the second drug affects the next phase of the cell cycle (Luszczki 2007; Berenbaum 1989). Our experience has shown that the most unfavorable interaction in human melanoma was the combination of cisplatin with docetaxel and cisplatin with selumetinib (a tendency towards antagonism in two of the four tested cell lines. This may be due to the fact that docetaxel, stabilizing microtubules, affects the G1/S phase (Goodson and Jonasson 2018) and blocks the action of cisplatin, which, by forming adducts with DNA, affects the replication phase (S) (Achkar et al. 2018). Selumetinib is an inhibitor of the MEK pathway (Najem et al. 2017 Nov), affects cell progression in the cycle, and can block the transition from G1 to S phase, which also excludes the action of cisplatin. The combination of cisplatin with docetaxel was successfully tested in patients with squamous cell carcinoma of the head and neck, in whom the administration of two drugs at the same time was well tolerated and had moderate efficacy (Kumai et al. 2022). However, cutaneous melanoma differs in its response to cytostatics from squamous cell carcinoma (Ma et al. 2020). In the case of melanoma cells, the adverse interaction may be due to an inappropriate drug delivery system, as demonstrated in B16F1 murine melanoma cells, where the combination of cisplatin with docetaxel administered via a polypeptide-based micelle system showed increased antitumor activity (Song et al. 2014). In the case of the combination of cisplatin with selumetinib, it was shown in phase II clinical trials that the administration of selumetinib in combination with the standard therapy of cisplatin with gemcitabine did not improve the efficacy and increased toxicity in patients with advanced biliary tract cancer (Doherty et al. 2022 Nov).

Additive interactions are most commonly observed. Additive interactions occur when a two-drug mixture inhibits the growth of cancer cells by adding up the effects of drugs used separately (Luszczki 2007; Berenbaum 1989). Our experiments on human melanoma cell lines have shown additive interactions in the case of combinations of mitoxantrone with selumetinib, docetaxel with mitoxantrone, and cisplatin with vemurafenib. Although the drugs affect other targets, such as mitoxantrone inhibiting DNA synthesis (Legut et al. 2014), selumetinib is the MEK inhibitor, and vemurafenib is the BRAF inhibitor (Najem et al. 2017 Nov).

The most desirable interaction is synergy, especially in oncology. In general, if two drugs synergistically inhibit proliferation, they probably affect different phases/sites of the cell cycle, ultimately contributing to a faster elimination of cancer cells (Luszczki 2007; Berenbaum 1989). The experiments showed that the most promising combination, which showed statistically significant synergy in the case of three of the four tested cell lines (in the case of the fourth, a tendency towards synergy), was the combination of vemurafenib with docetaxel. Vemurafenib is effective only in the case of cells with the BRAF mutation (all the cell lines tested herein have the BRAF mutation), selectively binds to the ATP binding site of the BRAF V600E kinase, and inhibits its activity, affecting the G1 phase of the cell cycle (Garbe and Eigentler 2018). Docetaxel has a similar target point, acting mainly on the G1/S point dependent on the p53 protein (Yasuhira et al. 2016), hence the presumed synergistic effect of this two-drug combination.

Also, the combination of cisplatin with mitoxantrone (synergy for two cell lines and a tendency towards synergy for the other two) showed a beneficial effect against melanoma. Both chemotherapeutics affect the genetic material DNA of cancer cells in different mechanisms, with the effect of cisplatin being independent of the cell cycle (Achkar et al. 2018; Legut et al. 2014). The combination of cisplatin and mitoxantrone has been successfully tested and used in patients with ovarian cancer (Husain et al. 1999; Topuz et al. 1998). The additive interaction with a tendency towards synergy was also characterized by the combinations of vemurafenib with mitoxantrone and vemurafenib with selumetinib (synergy for one cell line and a tendency towards synergy for the other three cell lines). Vemurafenib, as a BRAF inhibitor, affects the G1 phase of the cycle (Najem et al. 2017 Nov). It did not block the action, but even intensified the action of mitoxantrone, affecting the phase of DNA synthesis and transcription (S/G2) (Legut et al. 2014).

It is worth noting that none of the combinations we tested with vemurafenib showed the antagonistic interaction, and in fact, most of them showed either synergy or a tendency towards synergy. This may be due to the direct effect of the compound on the BRAF mutation in melanoma cell lines. The combination of vemurafenib with selumetinib, as expected, showed synergy for one cell line and a tendency towards synergy for the other three cell lines. The combination of BRAF inhibitors with MEK inhibitors is used in the treatment of melanomas with the BRAF V600E and V600K mutation, especially in the treatment of inoperable or metastatic melanoma (Czupryn and Cisneros 2017). This treatment blocks the MAPK pathway at two signaling points and may reduce skin toxicity associated with paradoxical reactivation of the MAPK pathway (Polkowska et al. 2016; Flaherty et al. 2012). Phase III clinical trials have confirmed that combined treatment with BRAF and MEK inhibitors delays the emergence of treatment resistance and side effects compared with BRAF inhibitor monotherapy (Long et al. 2014 Nov 13). The combination of vemurafenib and cobimetinib significantly improved progression-free survival and overall survival compared with vemurafenib alone (Long et al. 2014 Nov 13; Larkin et al. 2014). The combination of BRAF and MEK inhibitors also acts synergistically against melanoma cells with the NRAS mutation (due to higher activity of the MAPK pathway) (Atefi et al. 2015). Although treatment with BRAF/MEK inhibitors brings a number of benefits compared to monotherapy, it also carries the risk of side effects such as skin or eye irritation and cardiotoxicity (Czupryn and Cisneros 2017).

It was also noted that the combination of the SRC family kinase (SFK) inhibitor dasatinib with one of the BRAF or MEK inhibitors—dabrafenib, vemurafenib, or trametinib—showed synergistic interactions in many tested melanoma cell lines, also derived from patients (regardless of the presence of BRAF mutations in the case of isolated cells) (Close et al. 2021). This confirms our observation that vemurafenib in combination with other chemotherapeutics shows additive-to-synergistic interactions.

In the treatment of melanoma, new methods and drug combinations are sought. CLTA-4 blocking antibodies (e.g., ipilimumab) are added to BRAF and MEK inhibitors, thus combining chemotherapy with immunotherapy. BRAF/MEK inhibitors have good response rates but poor progression-free survival, while immunotherapy aims to achieve durable tumor control. However, this method requires further research (Luke and Ott 2013). A phase I trial combining vemurafenib and ipilimumab was stopped early due to several cases of grade 3–4 hepatitis (DePalo et al. 2022). Other studies in patients with advanced melanoma confirmed improved response rates and prolonged overall survival after combined administration of ipilimumab (anti-CTLA-4 antibody) with carboplatin or Taxol (Jamal et al. 2017).

Our in vitro experiments on four melanoma cell lines evaluating the nature of the pharmacodynamic interactions between various two different chemotherapeutics provide many clues as to potential trends in multidrug therapy. Our experience is limited by the lack of studies on wild melanoma cells—obtained from patients—and the lack of animal experiments. However, a huge advantage is the use of the isobolographic analysis method—the gold standard in the statistical assessment of the nature of interactions between drugs.

## Summary

The treatment of metastatic melanoma is still a subject of extensive research. Currently used methods include the combination of BRAF and MEK inhibitors and immunotherapy methods (anti-CTLA-4 and anti-PD1/PDL1 antibodies), which significantly improve standard treatment. Monotherapy does not bring long-term benefits due to drug resistance that appears after some time. For this reason, the best two-drug combinations are sought that would improve the long-term, durable response of patients and overcome drug resistance. It would also be good if the combination used caused fewer side effects than in monotherapy. Our experience has shown that the most promising drug combinations in melanoma therapy are vemurafenib with docetaxel, vemurafenib with mitoxantrone, vemurafenib with selumetinib, and cisplatin with mitoxantrone.

## Data Availability

All source data for this work (or generated in this study) are available upon reasonable request.
